# Epitranscriptomic control of cancer: the emerging roles of m⁵C and ac⁴C RNA modifications

**DOI:** 10.1038/s41420-026-03135-6

**Published:** 2026-05-12

**Authors:** Xian Zhong, Jianmei Mao, Jiawei Zhang

**Affiliations:** 1 Department of Medical Oncology, The Second Affiliated Hospital, School of Medicine, Zhejiang University, Hangzhou, China; 2 Cancer Institute (Key Laboratory of Cancer Prevention and Intervention China National Ministry of Education), The Second Affiliated Hospital, School of Medicine, Zhejiang University, Hangzhou, China; 3 Center for Genetic Medicine, The Fourth Affiliated Hospital, School of Medicine, Zhejiang University, Yiwu, China; 4 Institute of Genetics, International School of Medicine, Zhejiang University, Hangzhou, China

**Keywords:** Cancer, Cancer metabolism

## Abstract

Cytidine RNA modifications have emerged as key regulators of tumor cancer biology, linking transcriptional control to metabolic adaptation and immune evasion. Among them, 5-methylcytidine (m⁵C) and N⁴-acetylcytidine (ac⁴C) represent dynamic and functionally complementary epitranscriptomic marks that operate through distinct regulatory layers. m⁵C, catalyzed by the NSUN family methyltransferases, primarily stabilizes pro-tumorigenic transcripts, enhances glycolysis, and suppresses antitumor immunity through modulation of cytokine and checkpoint pathways. In parallel, ac⁴C, mediated by the acetyltransferase NAT10, fine-tunes translational efficiency and proteostasis, enabling tumor cells to adapt to metabolic and therapeutic stress. Together, these modifications cooperatively remodel the tumor immune microenvironment by driving macrophage polarization, T-cell exhaustion, and attenuation of interferon signaling, establishing a durable immunosuppressive niche. Notably, pharmacologic or genetic inhibition of m⁵C- and ac⁴C-modifying enzymes reverses malignant phenotypes and restores sensitivity to immune checkpoint and metabolic therapies. Elucidating this two-layer cytidine epitranscriptomic architecture unveils new epigenetic dimensions of tumor plasticity and offers promising avenues for precision RNA-targeted oncology.

## Facts

- Cytidine RNA modifications (m⁵C and ac⁴C) function as active regulatory layers, rather than passive chemical marks, integrating RNA stability, translation, metabolism, and immune signaling to shape tumor progression and therapy response.
- m⁵C and ac⁴C act through distinct yet complementary regulatory layers: m⁵C predominantly governs RNA stability and transcript fate, whereas ac⁴C primarily enhances translational efficiency and stress adaptation.
- Both modifications critically remodel the tumor immune microenvironment, promoting macrophage polarization, T-cell exhaustion, and suppression of interferon signaling, thereby contributing to immune checkpoint resistance.
- Pharmacologic or genetic disruption of m⁵C- and ac⁴C-modifying enzymes reverses malignant phenotypes in multiple tumor models, supporting the emerging druggability of the cytidine epitranscriptome.
- Cytidine RNA modification states correlate with tumor metabolic and immune phenotypes, positioning m⁵C/ac⁴C signatures as emerging biomarkers for prognosis and therapeutic stratification.

## Open questions

- Are m⁵C and ac⁴C causal drivers or adaptive amplifiers of tumor evolution? To what extent do these modifications initiate oncogenic programs versus stabilize pre-existing malignant states?
- How is modification specificity achieved across cell types and stress contexts? What determines selective targeting of transcripts by NSUN enzymes or NAT10 in tumor cells versus immune or stromal compartments?
- Can cytidine RNA modifications be selectively targeted without disrupting normal tissue homeostasis? Is it feasible to develop tumor- or context-specific inhibitors that decouple pathological RNA modification from physiological RNA metabolism?
- How do m⁵C and ac⁴C interact with other epigenetic and epitranscriptomic pathways? Do these modifications function hierarchically with m⁶A, chromatin marks, or metabolic epigenetic signals to coordinate tumor plasticity?
- Can temporal or sequential targeting of cytidine RNA modifications improve immunotherapy outcomes? Would priming tumors with m⁵C/ac⁴C inhibition before immune checkpoint blockade outperform concurrent combination strategies?

## Background

Epitranscriptomic regulation has emerged as a fundamental layer of post-transcriptional gene control, dynamically influencing RNA stability, translation, and metabolism across physiological and pathological contexts [1–3]. To date, more than 170 distinct RNA modifications have been identified, among which methylation and acetylation of cytidine residues play pivotal roles in shaping cellular function and disease progression [2–5]. These chemical marks fine-tune gene expression programs by modifying RNA structure and interactions with specific reader proteins, thereby orchestrating transcriptional plasticity and adaptive stress responses [1, 3]. Dysregulation of RNA modification enzymes has been increasingly recognized as a hallmark of cancer, contributing to malignant transformation, metabolic reprogramming, and immune evasion [6].

Among diverse epitranscriptomic marks, cytidine modifications—specifically 5-methylcytidine (m⁵C) and N⁴-acetylcytidine (ac⁴C)—represent two evolutionarily conserved yet mechanistically distinct forms of RNA modification. m⁵C is catalyzed primarily by the NSUN (NOP2/Sun RNA methyltransferase) family and DNMT2, while ac⁴C is deposited by the acetyltransferase NAT10^6–8^. These modifications occur across various RNA species, including tRNA, rRNA, mRNA, and noncoding RNA, exerting broad effects on RNA processing, export, translation, and degradation [7–9]. Importantly, m⁵C and ac⁴C are not merely structural marks but dynamic regulators that respond to metabolic cues and cellular stress, positioning them as critical integrators of oncogenic signaling and the tumor immune microenvironment (TME) [6, 7, 9].

Importantly, although numerous cytidine RNA modifications have been identified—including hm⁵C, f⁵C, and m¹C—this Review focuses on m⁵C and ac⁴C because they represent the two best-characterized and functionally dominant cytidine modifications in cancer, with well-defined enzymatic machineries and emerging therapeutic relevance. More critically, these two modifications operate at distinct yet complementary regulatory layers of RNA biology. m⁵C primarily governs RNA fate, including transcript stability, nuclear export, and decay, thereby shaping the abundance of oncogenic transcripts. ac⁴C predominantly regulates RNA output, enhancing translational efficiency, codon decoding, and proteostasis, particularly under conditions of metabolic or therapeutic stress. We therefore propose that m⁵C and ac⁴C together constitute a two-layer cytidine epitranscriptomic system, in which RNA abundance and protein output are coordinately regulated to enable tumor plasticity. This framework provides a conceptual basis for understanding how cytidine RNA modifications integrate metabolism, stress adaptation, and immune evasion.

In this review, we summarize recent progress in elucidating the molecular mechanisms of m⁵C and ac⁴C in cancer development and immune regulation. We discuss how these modifications influence key oncogenic pathways, remodel the tumor immune microenvironment, and mediate therapy resistance. Finally, we highlight emerging strategies targeting cytidine RNA modifications and their potential in improving the efficacy of anticancer treatments.

## m^5^C modification in cancer

### m⁵C machinery and regulatory architecture

Among the numerous epitranscriptomic marks, 5-methylcytidine (m⁵C) is one of the most conserved and intensively studied RNA modifications. The establishment, recognition, and removal of this modification are governed by a sophisticated network of enzymes collectively termed the “writers,” “readers,” and “erasers” [2]. Together, these components dynamically regulate RNA metabolism—including splicing, export, translation, and degradation—and thereby influence gene expression patterns within the tumor microenvironment [6, 9].

Writers catalyze the methylation of cytidine residues at the fifth carbon position [9, 10]. The primary enzymes include DNA methyltransferase homolog 2 (DNMT2, also known as TRDMT1) and members of the NOL1/NOP2/SUN domain (NSUN) family [2, 6, 10]. These methyltransferases introduce m⁵C marks across diverse RNA species to modulate RNA stability, translational efficiency, and degradation, ultimately shaping oncogenic transcriptional programs [8, 9, 11]. Remarkably, m⁵C writers display distinct subcellular localization: NSUN2, NSUN5–7, and NOP2 function predominantly in the nucleus, whereas NSUN2/NSUN3 and NSUN4 operate within mitochondria to modify tRNAs and 12S rRNA, respectively [10]. Among them, NSUN2 is unique in spanning the nucleus, ribosomes, and mitochondria, thereby linking compartment-specific m⁵C regulation [10].

Readers recognize and interpret m⁵C-modified transcripts, translating chemical marks into functional outcomes. Representative examples include ALYREF (Aly/REF export factor) and YBX1 (Y-box–binding protein 1), both of which are crucial for RNA export and stability. In esophageal squamous cell carcinoma, YBX1 binds and stabilizes m⁵C-modified *SMOX* mRNA, promoting malignant proliferation and migration [12]. Similarly, in colorectal cancer, ALYREF recruits ELAVL1 to m⁵C-tagged transcripts, enhancing nuclear export and transcript stability, thereby facilitating tumorigenesis [13].

Erasers mediate the removal or oxidation of m⁵C, maintaining a dynamic equilibrium within the RNA methylome. The TET (ten-eleven translocation) family and ALKBH1 have been implicated in this demethylation process [2, 6]. Notably, TET2-catalyzed oxidation of m⁵C generates 5-hydroxymethylcytidine (hm⁵C), a transient intermediate that regulates chromatin state and has been linked to leukemogenesis [14].

m⁵C sites are broadly distributed across coding and noncoding regions, typically enriched near translation start sites and 3′ untranslated regions (UTRs) [8, 9]. Their positioning allows fine-tuning of mRNA stability and translation efficiency [9, 15]. In physiological settings, m⁵C contributes to RNA quality control, mitochondrial function, and differentiation [10]. However, dysregulation of m⁵C methyltransferases or readers is frequently observed in cancer, underscoring their pivotal role in tumorigenesis.

### m⁵C-mediated regulation in tumor biology

#### Proliferation: m⁵C coordinates oncogenic growth and metabolic reprogramming

Accumulating evidence establishes m⁵C modification as a pivotal driver of tumor cell proliferation. Through the concerted actions of its writers and readers, m⁵C stabilizes and enhances translation of pro-growth transcripts while coupling these programs to metabolic rewiring that sustains anabolic demand.

NSUN2 serves as the central proliferative methyltransferase. Activated by diverse upstream signals, NSUN2 installs m⁵C marks on specific mRNAs encoding cell-cycle and metabolic regulators—including *c-MYC*, *PKM2*, *GRB2*, *HIP1*, *PIK3R1*, *PCYT1A* and *E2F1*—thereby promoting their stability and translational efficiency. In gastric cancer, SUMO-2/3-mediated modification of NSUN2 increases its activity and enhances methylation of pro-proliferative transcripts, including *PIK3R1* and *PCYT1A*,driving cell division [16]. In prostate cancer, NSUN2 forms a positive feedback loop with androgen receptor (AR) signaling to sustain proliferative transcriptional networks [17]. Similarly, in hepatocellular carcinoma, NOP2-mediated m⁵C modification of *c-MYC* mRNA augments EIF3A-dependent translation [18], while NSUN2-dependent methylation of *PKM2* reinforces glycolytic flux, together supporting rapid biomass synthesis [19]. In multiple myeloma, NSUN2 stabilizes *HIP1* mRNA via m⁵C deposition, and in esophageal squamous cell carcinoma [20], NSUN2-modified *GRB2* transcripts recruit LIN28B to amplify RTK–MAPK/PI3K signaling cascades that fuel proliferation [21].

The biological output of m⁵C modification largely depends on reader proteins that recognize and protect methylated transcripts. YBX1 exemplifies this mechanism: in esophageal squamous cell carcinoma, YBX1 binds m⁵C-modified *SMOX* mRNA to promote proliferation and migration, while in ovarian cancer it facilitates phase separation and stabilizes *E2F1* transcripts to sustain cell-cycle progression [12, 22]. ALYREF, often in cooperation with ELAVL1, binds m⁵C-tagged RNAs to enhance their nuclear export and stability, thereby driving tumorigenesis in colorectal and liver cancers [13, 23]. These findings underscore that m⁵C’s oncogenic effects emerge from a coordinated writer–reader axis that governs transcript fate.

Conversely, loss-of-function data highlight the essential nature of this system: global NSUN2 deletion induces translational rewiring incompatible with sustained proliferation [24]. Yet, not all NSUN family members act uniformly as oncogenes. NSUN6 displays a context-dependent suppressive function in pancreatic ductal adenocarcinoma, where its m⁵C deposition represses proliferation [25]. NSUN5 also exhibits a tumor-suppressive role in glioma—its epigenetic silencing abolishes 28S-C3782 methylation, triggering a stress-adaptive translational program that limits growth but sensitizes cells to NQO1-bioactivation therapy and correlates with favorable prognosis [26]. These examples emphasize that the biological consequences of m⁵C are highly context dependent, dictated by target transcript profiles and cellular environment. Clinical translation therefore requires stratification of patients based on the molecular configuration of the m⁵C regulatory network.

Beyond canonical RNA-stabilizing functions, m⁵C-modified transcripts also participate in genome maintenance and chromatin remodeling. During DNA-damage responses, TRDMT1-mediated m⁵C deposition promotes homologous recombination repair, supporting survival under genotoxic stress [15]. Moreover, m⁵C oxidation by TET2 to 5-hydroxymethylcytidine (hm⁵C) can remodel chromatin accessibility and transcriptional output, particularly in hematologic malignancies, thereby linking RNA methylation to epigenetic transcriptional control [14].

**Fig. 1:**
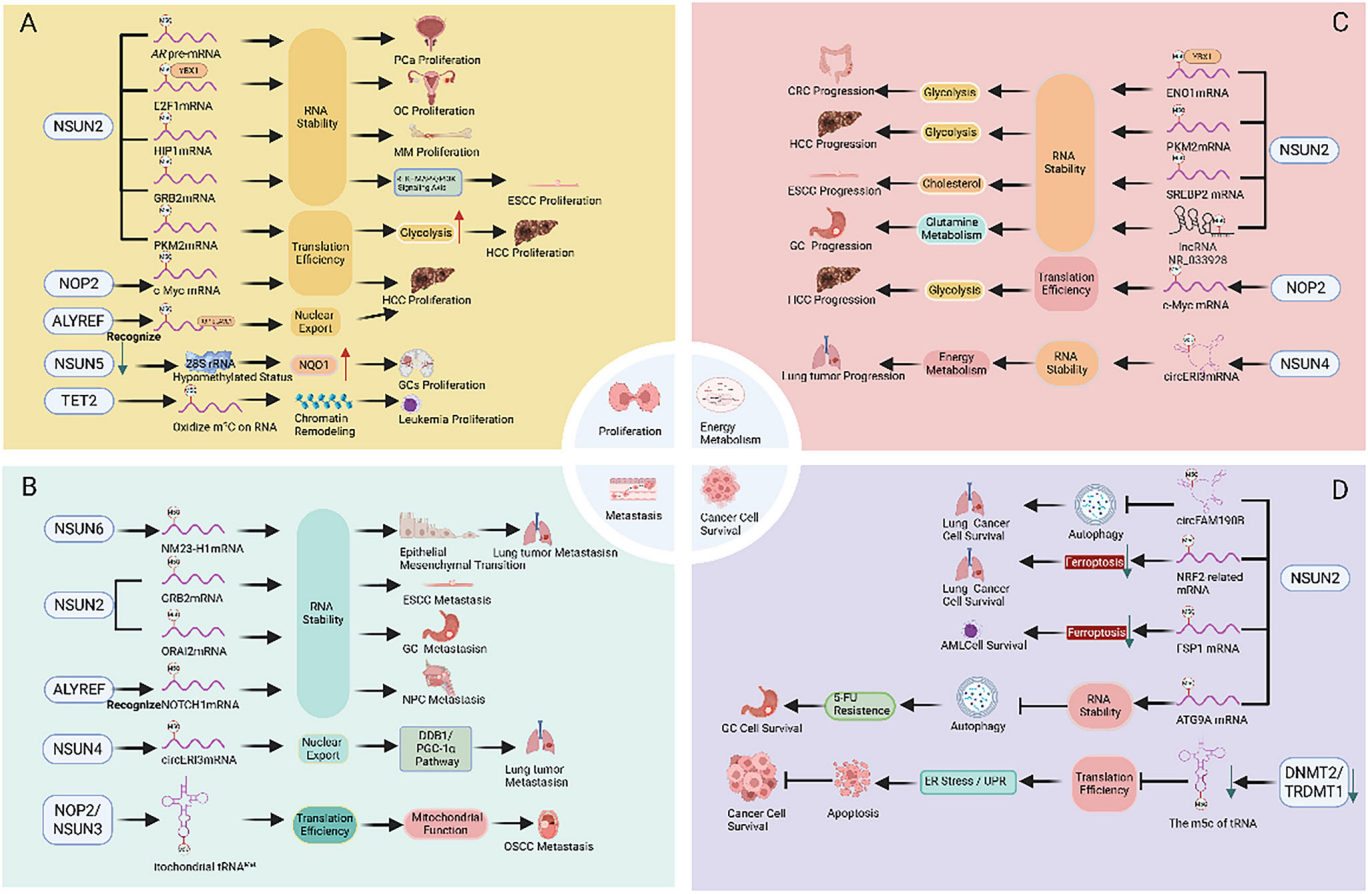
The multifaceted roles of RNA 5-methylcytidine (m⁵C) modification in tumor progression. **A** m⁵C-mediated regulation of tumor proliferation. m⁵C writers (e.g., NSUN2, NOP2) and readers (e.g., ALYREF) enhance RNA stability, translation efficiency, and nuclear export of oncogenic transcripts such as E2F1, MYC, GRB2, and PKM2. These processes activate proliferative signaling pathways (e.g., MAPK/PI3K) and metabolic programs (e.g., glycolysis), thereby promoting tumor growth across multiple cancer types. Additional layers include rRNA modification (NSUN5), RNA oxidation (TET2), and chromatin remodeling, which further support proliferation and genome stability. **B** m⁵C-mediated regulation of tumor metastasis. m⁵C modification promotes metastatic progression by stabilizing transcripts involved in epithelial–mesenchymal transition (EMT), cell migration, and invasion. NSUN2- and NSUN6-dependent m⁵C deposition enhances expression of metastasis-associated genes (e.g., NM23-H1, GRB2, ORAI2), while ALYREF-mediated RNA export and NSUN4-dependent mitochondrial regulation support dissemination and colonization. These processes converge on EMT activation and metabolic adaptation, facilitating metastatic spread in multiple tumor types. **C** m⁵C-driven metabolic reprogramming in cancer. m⁵C modification reprograms tumor metabolism by stabilizing key metabolic transcripts, including ENO1, PKM2, SREBP2, and c-MYC. This promotes glycolysis, cholesterol biosynthesis, glutamine metabolism, and mitochondrial energy production. Through coordinated regulation of RNA stability and translation efficiency, m⁵C enables metabolic plasticity that supports tumor growth under varying environmental conditions. **D** m⁵C-mediated regulation of cancer cell survival and stress adaptation. m⁵C modification enhances tumor cell survival by modulating multiple cell death and stress-response pathways. These include suppression of ferroptosis (via NRF2 and FSP1), regulation of autophagy (e.g., ATG9A), and control of ER stress/UPR signaling. Additionally, m⁵C supports DNA damage repair and chemoresistance (e.g., 5-FU resistance), enabling cancer cells to withstand therapeutic and microenvironmental stress.

In summary, m⁵C modification—predominantly through NSUN2 and its reader network—constitutes a central epigenetic circuit sustaining malignant proliferation (Fig. 1A). It stabilizes and enhances translation of key proliferative transcripts, integrates metabolic states, and connects RNA metabolism with DNA repair and chromatin activity. Nevertheless, most evidence derives from correlation or overexpression studies; future research should establish site-specific causal mechanisms, delineate reader selectivity, and develop precise inhibitors to safely modulate this pathway. Because YBX1, ALYREF, and other readers also participate in overlapping RNA-regulatory systems such as the m⁶A network, dissecting m⁵C-specific effects will be critical for rational therapeutic targeting. Notably, although many studies support a tumor-promoting role for m⁵C, the current evidence more consistently supports a model in which m⁵C acts as a state-stabilizing amplifier of proliferative signaling, rather than a universal initiating driver across tumor types.

#### Metastasis: m⁵C regulates EMT, metabolic adaptation, and invasive behavior

Beyond promoting proliferation, m⁵C modification critically orchestrates tumor invasion and metastasis by coupling epithelial–mesenchymal transition (EMT), metabolic reprogramming, and microenvironmental adaptation. These effects collectively endow tumor cells with the plasticity required for dissemination, colonization, and metastatic outgrowth.

##### m⁵C-dependent regulation of EMT and signaling networks

Several studies demonstrate that aberrant m⁵C deposition reshapes EMT-associated transcriptional programs. In lung cancer, NSUN6-mediated m⁵C modification of *NM23-H1* alters EMT marker expression and enhances invasive potential [27]. In head and neck squamous cell carcinoma (HNSCC), an m⁵C-based EMT-associated prognostic signature correlates with aggressive phenotypes, underscoring the strong link between m⁵C-regulated transcripts and metastatic progression [28]. In esophageal squamous cell carcinoma (ESCC), NSUN2-catalyzed m⁵C on *GRB2* mRNA enables LIN28B-dependent stabilization, amplifying RTK–MAPK/PI3K signaling to drive cell migration and invasion [21]. In nasopharyngeal carcinoma, ALYREF-mediated stabilization of m⁵C-modified NOTCH1 mRNA activates NOTCH signaling, thus promoting metastatic progression [29]. Likewise, in hepatocellular carcinoma (HCC), loss of 5-hydroxymethylcytidine (5hmC)—a TET-oxidized derivative of m⁵C—activates the TET–SOCS1–MMP9 axis and promotes metastasis, suggesting mechanistic parallels between m⁵C/TET pathways and invasive behavior [30].

##### Integration with metabolic and mitochondrial dynamics

Metastatic dissemination demands profound metabolic flexibility, and m⁵C-mediated RNA regulation contributes directly to this adaptation. In Oral cancer, mitochondrial RNA methylation, including m⁵C modification, supports oxidative metabolism and energy reallocation during metastasis [31]. In lung cancer, NSUN4-dependent m⁵C of *circERI3* modulates mitochondrial bioenergetics, thereby facilitating metastatic competence [32]. In gastric cancer, fatty acid supply from peritoneal adipocytes triggers AMPK–E2F1–NSUN2 signaling, leading to NSUN2-mediated m⁵C activation of ORAI2 and promoting peritoneal dissemination and colonization [33]. These findings reveal that m⁵C not only modulates cytoplasmic mRNA translation but also governs mitochondrial RNA networks that drive metabolic resilience.

##### Non-cell-autonomous and microenvironmental roles

Emerging evidence indicates that m⁵C exerts effects beyond tumor-intrinsic signaling. m⁵C-modified exosomal RNAs participate in intercellular communication, influencing tumor-associated macrophage (TAM) polarization and shaping a permissive metastatic niche [34]. Such m⁵C-dependent exosome signaling likely contributes to the “containment–transfer” dynamic within the pre-metastatic microenvironment [34]. These observations highlight m⁵C as a regulator not only of tumor cell motility but also of systemic adaptation to metastatic stress.

In summary, m⁵C modification functions as a full-spectrum regulator of metastatic progression (Fig. 1B), acting through three interconnected layers: 1. Transcriptional and post-transcriptional control of EMT and signaling cascades (e.g., *NM23-H1*, *GRB2* → MAPK/PI3K) [21, 27–30]. 2. Metabolic and mitochondrial reprogramming that sustains energy production and redox balance during dissemination [31–33]. 3. Microenvironmental remodeling via exosomal m⁵C signaling that modulates immune and stromal components [34]. Its influence spans every metastatic stage—from local invasion and intravasation to distant colonization—reflecting a reversible, context-dependent adaptation rather than a mere extension of proliferative signaling. The ultimate outcome of m⁵C-mediated regulation depends on the spectrum of modified targets and the expression of specific reader proteins such as YBX1, ALYREF, and ELAVL1 [13, 22, 32].

#### Metabolism: m⁵C reprograms tumor energy and biosynthetic metabolism

Metabolic reprogramming is a hallmark of cancer, allowing tumor cells to sustain rapid proliferation and adapt to nutrient or oxidative stress. Recent studies reveal that m⁵C RNA modification integrates transcriptional, translational, and metabolic control to coordinate energy production and biosynthetic pathways. By acting on both nuclear-encoded and mitochondrial transcripts, m⁵C serves as a critical epitranscriptomic regulator of glycolysis, oxidative phosphorylation (OXPHOS), and macromolecule synthesis.

##### Mitochondrial m⁵C modifications govern OXPHOS and TCA cycle efficiency

Emerging evidence demonstrates that m⁵C modification extends beyond cytoplasmic RNAs to the mitochondrial transcriptome, where it regulates oxidative metabolism and the tricarboxylic acid (TCA) cycle. Delaunay et al. showed that metastasis-associated RNA modifications, including m⁵C, remodel mitochondrial activity to enhance metabolic plasticity and energy flexibility [31]. Similarly, Wu et al. identified that NSUN4-mediated m⁵C modification of *circERI3* modulates mitochondrial respiration in lung cancer, promoting tumor growth and metastatic potential [32].

These findings challenge the traditional view of m⁵C as a purely cytoplasmic mark and suggest that it functions as a “mitochondrial switch” enabling cancer cells to adapt to fluctuating oxidative and energetic demands. Through this mechanism, m⁵C promotes tumor survival under chemotherapy, oxidative stress, and hypoxia, providing a plausible basis for metabolic resilience in treatment-resistant cancers.

##### m⁵C stabilizes glycolytic enzyme transcripts and reinforces the Warburg effect

In addition to its mitochondrial role, m⁵C directly regulates glycolytic metabolism by stabilizing mRNAs encoding key rate-limiting enzymes. In colorectal cancer (CRC), NSUN2 and YBX1 form a positive feedback loop in which YBX1 recognizes m⁵C-modified *ENO1* mRNA, enhancing its stability and glycolytic output [35]. In hepatocellular carcinoma (HCC), NSUN2 increases the expression and activity of *PKM2*, driving aerobic glycolysis and anabolic growth [19].

Furthermore, the HIF-1α–ALYREF–PKM2 axis integrates hypoxic signaling with m⁵C-mediated mRNA stabilization [36], while elevated glucose levels can directly enhance NSUN2 activity, reinforcing glycolytic translation [37]. Collectively, these findings define m⁵C as an active metabolic regulator that selectively stabilizes glycolytic transcripts rather than globally altering transcriptional programs.

Therapeutically, targeting m⁵C may represent a more refined strategy than direct inhibition of glycolytic enzymes such as PKM2. Because metabolic inhibitors often cause systemic toxicity, m⁵C blockade could achieve similar outcomes with fewer side effects and may synergize with glucose restriction or metabolic pathway inhibitors. Importantly, m⁵C-driven lactate accumulation and acidification also promote immune suppression in the tumor microenvironment, linking metabolic control to tumor–immune interactions.

##### m⁵C-dependent regulation of lipid and glutamine metabolism supports biosynthetic demands

Beyond energy production, m⁵C coordinates broader biosynthetic programs essential for tumor progression. In hepatocellular carcinoma, NOP2-mediated m⁵C promotes *c-MYC*-dependent metabolic reprogramming that couples glucose utilization with lipid and glutamine biosynthesis [18]. In gastric cancer, the m⁵C-modified lncRNA NR_033928 stabilizes *GLS* mRNA, enhancing glutaminolysis and supporting proliferation [38]. Similarly, in esophageal squamous cell carcinoma (ESCC), TTPAL amplification increases NSUN2-mediated m⁵C on *SREBP2* mRNA, upregulating cholesterol biosynthesis and membrane synthesis [39].

These examples indicate that m⁵C orchestrates the post-transcriptional control of multiple metabolic nodes, ensuring that tumor cells maintain adequate substrates for energy and biomass production. However, most current studies focus on individual enzymes, leaving the systems-level architecture of m⁵C-mediated metabolic rewiring unresolved. Future integrative approaches combining metabolomics, ribosome profiling, and epitranscriptomics will be essential to delineate how m⁵C synchronizes multiple biosynthetic pathways and interfaces with oncogenic signaling networks. Such understanding may uncover novel metabolic vulnerabilities for targeted intervention.

In summary, m⁵C modification represents a central epitranscriptomic mechanism coupling RNA fate with metabolic homeostasis (Fig. 1C). By stabilizing glycolytic, mitochondrial, and biosynthetic transcripts, m⁵C enables tumors to balance energy generation and anabolic growth. These functions not only sustain proliferation and metastasis but also promote immune evasion through metabolic–immune crosstalk. Deciphering the full scope of m⁵C-driven metabolic regulation may provide new opportunities to disrupt the metabolic foundation of cancer.

#### Cell survival and death resistance: m⁵C regulation of autophagy, apoptosis, and ferroptosis

Cancer cells continuously balance survival and death signals to endure metabolic, therapeutic, and microenvironmental stress. Mounting evidence indicates that m⁵C RNA modification functions as a pivotal regulator of this balance, rewiring post-transcriptional networks that govern autophagy, apoptosis, and ferroptosis. By stabilizing or selectively modulating the translation of death-related transcripts, m⁵C enables tumor cells to resist cytotoxic stress and maintain viability under adverse conditions.

##### m⁵C-mediated control of autophagy enables cytoprotective persistence

Autophagy is a double-edged process in cancer—suppressing tumor initiation yet sustaining established tumors under stress [40, 41]. Increasingly, it is recognized as a key contributor to therapeutic resistance [40]. Mechanistically, m⁵C modification interfaces with autophagy pathways to fine-tune autophagic flux, converting potentially lethal stress into adaptive survival responses.

In gastric cancer, the m⁵C reader YBX1 binds and stabilizes m⁵C-modified *ATG9A* transcripts, thereby regulating autophagic activity and promoting resistance to 5-fluorouracil (5-FU)–induced cytotoxicity [42]. Conversely, in lung cancer, NSUN2-mediated m⁵C modification of *circFAM190B* represses autophagy, preserving cellular integrity and promoting stress adaptation [43]. These findings demonstrate that m⁵C acts as a context-dependent modulator of autophagy, dynamically toggling between activation and suppression to sustain tumor survival during chemotherapy and nutrient deprivation.

##### m⁵C modulates apoptosis and the DNA damage response

m⁵C modification also governs apoptotic signaling by integrating endoplasmic reticulum (ER) stress, unfolded protein response (UPR) regulation, and DNA damage repair. Loss of the tRNA methyltransferase DNMT2/TRDMT1 lowers the UPR tolerance threshold, triggering ER stress–induced apoptosis [44]. In parallel, m⁵C deposition on DNA double-strand break (DSB)-responsive transcripts enhances their stability and translation, promoting homologous recombination repair and protecting cells from genotoxic injury [15]. Through these mechanisms, m⁵C functions as a “damage-encoded survival code”, reinforcing DNA repair capacity while attenuating apoptotic execution under stress.

##### m⁵C protects against ferroptosis by maintaining redox homeostasis

Beyond classical apoptosis, m⁵C modification safeguards tumor cells from ferroptosis, a non-apoptotic, lipid peroxidation–driven form of cell death. In non–small cell lung cancer (NSCLC), NSUN2 activation enhances NRF2-dependent antioxidant defense, preventing ferroptotic collapse under oxidative stress [45]. Similarly, in acute myeloid leukemia (AML), NSUN2-mediated m⁵C stabilization of *FSP1* mRNA augments lipid repair and redox balance, protecting leukemic cells from ferroptotic injury [46]. These findings reveal that m⁵C-mediated transcript stabilization not only preserves antioxidant capacity but also reinforces the lipid homeostasis required for survival under metabolic and therapeutic stress.

Collectively, these studies define m⁵C as a stress-conditional survival switch that dynamically rewires cellular death checkpoints (Fig. 1D). By modulating autophagy, apoptosis, and ferroptosis through selective transcript stabilization, m⁵C establishes a death-refractory state that underlies therapeutic resistance in diverse malignancies. Future work should elucidate how distinct m⁵C-regulated survival pathways interact within specific stress contexts, and whether pharmacologic disruption of this network can restore tumor cell vulnerability to conventional therapies.

#### Therapy resistance: m⁵C as an adaptive amplifier of treatment tolerance

Emerging evidence indicates that m⁵C modification contributes to therapy resistance not only by sustaining basal malignant programs but also by enabling adaptive survival under treatment-induced stress. Mechanistically, m⁵C enhances therapeutic tolerance through several non-mutually exclusive routes, including DNA damage repair, stress-responsive translation, metabolic buffering, and immune escape.

One prominent example is TRDMT1/DNMT2-mediated m⁵C deposition at DNA damage-associated transcripts, which promotes homologous recombination repair and preserves genome integrity under genotoxic stress [16]. This suggests that m⁵C can function as a damage-adaptive survival code, allowing tumor cells to better withstand chemotherapy or radiotherapy. In parallel, m⁵C-dependent stabilization of proliferative and metabolic transcripts—such as PKM2, ENO1, and c-Myc-related targets—supports continued energy production and biosynthetic output during therapeutic challenge [20, 21, 36].

m⁵C also intersects with cell death resistance pathways, including autophagy, ER stress tolerance, and ferroptosis suppression, thereby reinforcing persistence under treatment pressure [41–47]. At the immune level, m⁵C-dependent suppression of cGAS–STING–IFN signaling and promotion of T-cell dysfunction may further contribute to resistance to immune checkpoint blockade [48, 49].

Collectively, these findings support a model in which m⁵C acts less as a uniform initiating lesion and more often as a treatment-adaptive amplifier, stabilizing malignant cell states and buffering stress responses that permit therapeutic escape.

### m^5^C and tumor immune microenvironment

The tumor immune microenvironment (IME) represents a dynamic battleground where metabolic, epigenetic, and immunologic circuits converge. Mounting evidence reveals that m⁵C RNA modification acts as a central coordinator of this landscape, enabling tumors to evade immune surveillance by reprogramming both innate and adaptive immunity. Through coordinated regulation of interferon signaling, myeloid differentiation, T-cell exhaustion, and immune checkpoint expression, the m⁵C machinery establishes a multilayered scaffold of immune resistance.

#### m⁵C reprograms immune cell populations and promotes immune escape

Across diverse tumor types, m⁵C writers and readers operate as potent immune-escape actuators. Their activity can be mapped to four principal intervention layers: Suppression of innate immune signaling (e.g., IFN and cGAS–STING pathways), Remodeling of myeloid compartments (macrophage repolarization and dendritic-cell recruitment), Induction of T-cell exhaustion, and Reinforcement of immune-checkpoint–mediated suppression [47, 48].

In colorectal cancer (CRC), m⁵C dampens interferon-driven cytotoxic responses, while NSUN2 directly suppresses the cGAS–STING axis, attenuating antitumor immunity and diminishing responsiveness to immune checkpoint blockade [47, 48]. Similarly, in luminal breast cancer, YBX1-linked CD8⁺ T-cell exhaustion synergizes with TAM infiltration to form a cooperative adaptive immunosuppressive circuit [49].

Consistent with these findings, loss of the m⁶A reader YTHDF1 restores dendritic-cell recruitment and antigen presentation capacity, indicating that RNA-modification–dependent defects in myeloid priming constitute a rate-limiting step for effective immune activation [50]. Extending this network into the metabolic–epigenetic–immune axis, NSUN2-mediated stabilization of *NEO1* mRNA in renal cell carcinoma (RCC) rewires glycolysis and drives histone lactylation, which in turn activates the MYC–POM121–PD-L1 cascade [51]. NSUN2 depletion reverses this program, lowering lactate-induced PD-L1 expression and reinstating CD8⁺ T-cell cytotoxicity with enhanced TNF-α infiltration [52]. Collectively, these data delineate a hierarchical m⁵C-dependent immune-escape network spanning innate sensing, myeloid modulation, and adaptive exhaustion.

Notably, not all m⁵C events are immunosuppressive. In colorectal cancer (COAD), Chen et al. revealed a counterregulatory mechanism in which NSUN5-mediated m⁵C modification of GPX4 mRNA enhances transcript stability and sustains redox equilibrium [53]. This modification preserves mitochondrial integrity and facilitates the activation of the cGAS–STING axis, thereby amplifying type I interferon signaling and promoting immune-cell infiltration. Similarly, In bladder cancer, NSUN6-mediated m⁵C methylation upregulates HDAC10 expression by enhancing its mRNA stability, thereby suppressing the transcription of macrophage-associated chemokines and limiting the recruitment of M2-polarized macrophages [54]. Such findings underscore that, within specific metabolic–epigenetic contexts, m⁵C can also reinforce innate immune sensing and antitumor immunity, functioning as a tumor-suppressive epitranscriptomic program rather than an immune-escape driver.

Importantly, m⁵C-driven immunosuppression does not simply mark the terminal stage of immune dysfunction. Instead, it selectively impairs key “interface nodes” within the antitumor cascade—interferon production, cytosolic DNA sensing, antigen presentation, and effector T-cell persistence—thereby establishing a covert yet durable immune-evasion phenotype. This mechanistic architecture explains why m⁵C-associated immunotherapy resistance is often structural and persistent rather than transient or stochastic.

#### Metabolic coupling: m⁵C links tumor metabolism to immune regulation

Recent studies highlight a reciprocal link between m⁵C-driven metabolism and immune suppression. Here, m⁵C does more than enhance metabolic flux—it converts metabolic signals into stable, heritable immunoregulatory programs. In colorectal cancer, the NSUN2–m⁵C–ENO1 feedback loop amplifies glycolytic flux, ensuring sustained metabolic support for tumor evolution [35]. In RCC, NSUN2-dependent histone lactylation demonstrates how metabolic by-products can be epigenetically recorded as immune-suppressive memory, contributing to “immune-cold” phenotypes and checkpoint resistance [51].

Thus, m⁵C acts as a metabolic–epigenetic translator, forming an integrated “m⁵C → metabolism → epigenetics → immune” circuit. Whether targeted disruption of this circuit can convert immune-cold tumors into immune-hot phenotypes remains a key unanswered question and a priority for future immuno-sensitization strategies.

#### m⁵C as a biomarker in tumor immunology

Beyond mechanistic roles, m⁵C-associated immune remodeling carries substantial prognostic and predictive value across cancers. In colorectal cancer (CRC), Chen et al. identified three distinct m⁵C modification phenotypes characterized by divergent tumor immune microenvironments—immune-desert, immune-inflamed, and immune-excluded—each displaying unique immune-cell infiltration profiles, checkpoint expression patterns, and survival outcomes [55]. An m⁵C-score derived from these regulators accurately predicted prognosis and response potential to immune checkpoint blockade. In pancreatic adenocarcinoma (PAAD), Yun et al. established an m⁵C-score reflecting regulatory methylation patterns that independently predict survival; high m⁵C-score tumors exhibit immune exclusion, reduced CD8⁺ T-cell infiltration, and elevated PD-L1 expression, signifying immunotherapy resistance [56].

In head and neck squamous cell carcinoma (HNSCC), a combined m⁶A/m⁵C/m¹A-related lncRNA signature correlates closely with immune infiltration and clinical outcome, underscoring the value of multi-modification RNA–immune networks for patient stratification [57]. Similarly, in colorectal cancer (CRC), Song et al. developed an m⁶A–m⁵C lncRNA signature that predicts immune infiltration, checkpoint expression, and prognosis [58]. In lung squamous cell carcinoma (LUSC), an m⁵C regulatory-factor signature delineates distinct immune-infiltration landscapes, checkpoint profiles, and prognostic outcomes [59]. Similarly, in prostate cancer (PRAD), m⁵C modification subtypes associate with immune-microenvironment phenotypes and biochemical-recurrence (BCR) risk, forming a “m⁵C–TME–immune-marker–recurrence” framework for precision prognostics [60].

**Fig. 2:**
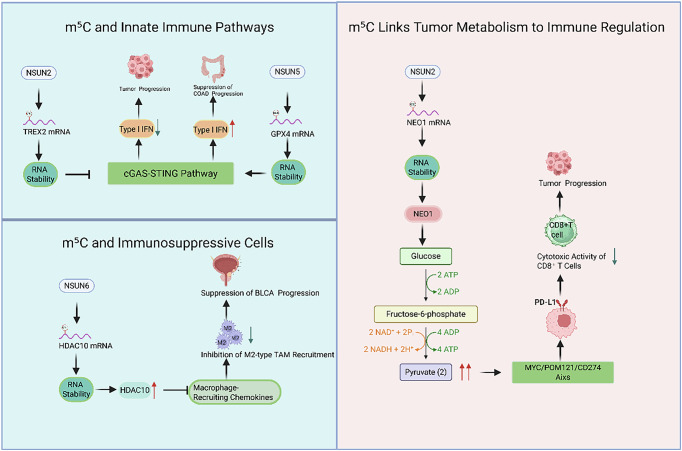
The role of RNA 5-methylcytidine (m⁵C) modification in shaping the tumor immune microenvironment. (Left, upper panel) m⁵C regulation of innate immune signaling pathways. m⁵C modification modulates innate immune responses primarily through regulation of the cGAS–STING pathway and type I interferon (IFN) signaling. NSUN2-mediated m⁵C stabilization of TREX2 mRNA promotes degradation of cytosolic DNA, thereby suppressing cGAS–STING activation and reducing type I IFN responses, which facilitates tumor progression. In parallel, NSUN5-dependent stabilization of transcripts such as GPX4 influences oxidative stress responses and may indirectly modulate innate immune signaling. These mechanisms collectively attenuate innate immune surveillance and support tumor growth. (Left, lower panel) m⁵C regulation of immunosuppressive cells in the tumor microenvironment. m⁵C modification shapes the tumor immune microenvironment by regulating tumor-associated macrophage (TAM) recruitment and polarization. NSUN6-mediated stabilization of HDAC10 mRNA increases HDAC10 expression, suppressing macrophage-recruiting chemokines and limiting M2-type TAM infiltration, thereby influencing tumor progression. This highlights a context-dependent role of m⁵C in modulating immunosuppressive cell populations within tumors. (Right panel) m⁵C links tumor metabolism to immune regulation. m⁵C modification integrates metabolic reprogramming with immune evasion. NSUN2-mediated stabilization of NEO1 mRNA enhances glycolytic flux, promoting conversion of glucose to pyruvate and increasing metabolic output. This metabolic shift activates the MYC/POM121/CD274 (PD-L1) axis, leading to elevated PD-L1 expression and suppression of CD8⁺ T-cell cytotoxic activity, thereby facilitating tumor progression. This panel illustrates how m⁵C-dependent metabolic remodeling translates into immunosuppressive signaling.

Together, these findings demonstrate that m⁵C not only orchestrates immune escape mechanistically but also serves as a robust biomarker of immune state and clinical trajectory. Integrating m⁵C-based metrics into immunogenomic profiling could refine patient selection for immunotherapy and reveal new vulnerabilities for epitranscriptomic intervention (Fig. 2).

## ac^4^C modification in cancer

### ac⁴C machinery and regulatory architecture

N⁴-acetylcytidine (ac⁴C) is a post-transcriptional RNA modification characterized by the acetylation of cytidine at the N⁴ position. Although first described in the early 2000s, it has since emerged as a critical epitranscriptomic mark regulating RNA stability, translation, and gene expression. ac⁴C is present in multiple RNA species—including tRNA, rRNA, mRNA, and even precursor miRNAs—highlighting its broad regulatory reach across gene-expression layers [7, 61].

To date, N-acetyltransferase 10 (NAT10) is the only identified “writer” enzyme responsible for ac⁴C modification in eukaryotes [61]. In contrast to m⁵C, no bona fide reader or eraser proteins have yet been conclusively identified for ac⁴C [62, 63]. This suggests either that ac⁴C is interpreted through noncanonical recognition mechanisms or that its biological effects arise largely through intrinsic changes in RNA structure, stability, or translational behavior. NAT10 is a multifunctional nucleolar acetyltransferase containing both a catalytic domain and lysine-rich RNA-binding regions that enable it to directly transfer an acetyl group from acetyl-CoA to cytidine residues [61, 64]. The reaction consumes acetyl-CoA and ATP, linking ac⁴C deposition to cellular metabolic state and energetic flux [65, 66].

Historically, ac⁴C was first identified on tRNAs and rRNAs in the 1960s–1970s [67, 68]. In tRNAs, ac⁴C modifications on tRNA^Met, as well as on the D-arms of tRNA^Ser and tRNA^Leu, enhance translational fidelity and thermal stability under stress, particularly under stress conditions [66, 69–71]. In mammalian ribosomes, NAT10-mediated acetylation of 18S rRNA reinforces ribosomal decoding accuracy, supporting the concept that ac⁴C is not merely a static structural mark but a regulated determinant of translational precision [61].

A major conceptual advance came with the discovery that ac⁴C also modifies mRNAs, where it exerts direct effects on mRNA stability and protein output. Transcriptome-wide mapping studies revealed that ac⁴C is enriched within coding sequences and near poly(A)-proximal regions, where it prolongs mRNA half-life and enhances translational efficiency [7]. Functionally, ac⁴C within coding sequences appears to facilitate ribosomal elongation, while ac⁴C in 5’ untranslated regions may modulate translation initiation. Genetic ablation of NAT10 eliminates mRNA-ac⁴C marks, resulting in reduced mRNA stability and global translational attenuation [7].

Beyond mRNAs, ac⁴C also participates in noncoding RNA regulation. Zhang et al. revealed that NAT10-dependent ac⁴C modification of pri-miRNAs enhances Drosha/DGCR8 processing, promoting maturation of oncogenic miRNAs and contributing to cancer progression [72]. Conversely, Yang et al. found that NAT10-mediated ac⁴C stabilization of *RUNX2* mRNA drives osteogenic differentiation and protects against bone loss, demonstrating that ac⁴C functions in both pathological and physiological contexts [73].

Collectively, these findings establish ac⁴C as a multilayered regulatory system that coordinates translational fidelity, mRNA stability, and RNA processing. Conceptually, whereas m⁵C more often acts at the level of RNA fate, ac⁴C appears to operate at a complementary layer that governs RNA output and translational efficiency. In cancer, dysregulation of the NAT10–ac⁴C axis therefore has the potential to enhance not only oncogenic protein synthesis but also the stress-adaptive and proteostatic capacity required for tumor progression and treatment tolerance.

### ac^4^C-mediated regulation in tumor biology

#### Proliferation: ac⁴C amplifies translational output and oncogenic growth

NAT10, the sole identified ac⁴C “writer,” has emerged as a direct amplifier of tumor cell proliferation through its dual regulation of tRNA and mRNA acetylation. Acting beyond transcriptional control, NAT10 enhances the translational landscape of oncogenic programs, effectively decoupling proliferative drive from genetic mutations or transcriptional activation.

Two primary layers of control have been delineated. In esophageal squamous cell carcinoma (ESCC), Wei et al. demonstrated that NAT10-driven ac⁴C modification of tRNAs enforces codon-biased translation, preferentially decoding EGFR-enriched codons and enabling elevated EGFR protein synthesis independent of transcriptional upregulation or genomic amplification [74]. This mechanism represents a translation-level gain of function supporting oncogenic output.

In a complementary pathway, Long et al. reported that NAT10 installs ac⁴C marks on proliferation-associated mRNAs, such as *HNRNPUL1*, increasing their stability and translational yield to maintain high proliferative rates [75]. Similarly, in gastric cancer (GC), Chen et al. revealed that NAT10-mediated ac⁴C modification of TNC mRNA enhances its stability and translation, leading to TNC overexpression that activates FAK/AKT/MAPK signaling, establishing a self-sustaining proliferative circuit [76].

**Fig. 3:**
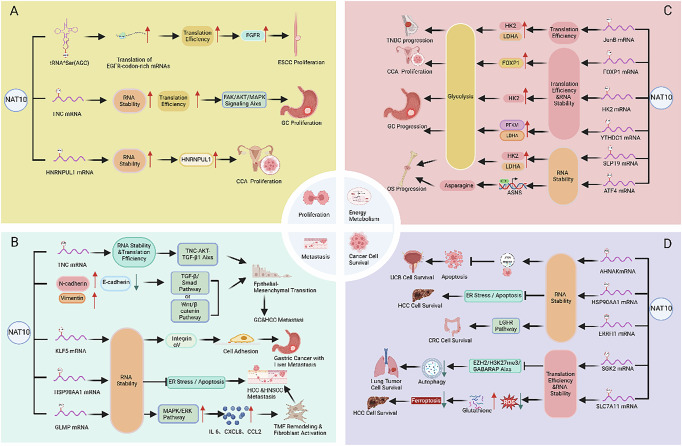
The multifaceted roles of RNA N⁴-acetylcytidine (ac⁴C) modification in tumor progression. **A** ac⁴C-mediated regulation of tumor proliferation. The ac⁴C writer NAT10 enhances tumor cell proliferation through dual mechanisms involving codon-biased translation and mRNA stabilization. ac⁴C modification of tRNAs promotes preferential translation of codon-enriched oncogenic transcripts such as EGFR, thereby increasing translational efficiency and driving proliferation in cancers such as ESCC. In parallel, NAT10-mediated ac⁴C deposition on mRNAs (e.g., TNC, HNRNPUL1) enhances RNA stability and protein output, activating proliferative signaling pathways (e.g., FAK/AKT/MAPK) and promoting tumor growth. **B** ac⁴C-mediated regulation of tumor metastasis. ac⁴C modification promotes metastatic progression by regulating epithelial–mesenchymal transition (EMT), cell adhesion, and tumor microenvironment remodeling. NAT10-dependent stabilization of transcripts such as TNC, KLF5, HSP90AA1, and GLMP activates EMT-associated pathways (e.g., TGF-β/SMAD, Wnt/β-catenin), enhances integrin-mediated adhesion, and induces secretion of cytokines (e.g., IL-6, CXCL8, CCL2) that promote fibroblast activation and stromal remodeling. These processes collectively facilitate invasion, dissemination, and metastatic colonization. **C** ac⁴C-driven metabolic reprogramming in cancer. NAT10-mediated ac⁴C modification reprograms tumor metabolism by stabilizing and enhancing translation of key metabolic regulators. ac⁴C-marked transcripts such as JunB, FOXP1, HK2, LDHA, YTHDC1, SIRT1, and ATF4 drive glycolysis, asparagine biosynthesis, and broader metabolic adaptation. These effects increase energy production, support biosynthetic demands, and contribute to tumor progression across multiple cancer types, including TNBC, cervical cancer, gastric cancer, and osteosarcoma. **D** ac⁴C-mediated regulation of cancer cell survival and therapy resistance. ac⁴C modification supports tumor cell survival by coordinating stress response pathways and suppressing cell death. NAT10 stabilizes transcripts such as HSP90AA1, ERH, SGK2, and SLC7A11, thereby modulating ER stress/UPR signaling, autophagy, and ferroptosis resistance through maintenance of redox balance and glutathione levels. Additionally, ac⁴C-dependent regulation of apoptosis and stress adaptation enhances resistance to therapeutic stress, positioning NAT10 as a central regulator of survival under adverse conditions.

Together, these findings identify ac⁴C as a protein-output–centric proliferative axis—a post-transcriptional mechanism that bypasses classical genomic and transcriptional constraints, enabling rapid malignant expansion and adaptation under selective pressure (Fig. 3A). These observations suggest that ac⁴C does not simply initiate proliferation, but frequently enhances the efficiency and resilience of oncogenic protein output, particularly in tumors already under high translational demand.

#### Metastasis: ac⁴C reprograms EMT, invasion, and tumor–stroma interactions

Accumulating evidence demonstrates that NAT10-mediated ac⁴C plays a pivotal role in metastasis through a coordinated, multi-layered program that integrates epithelial–mesenchymal transition (EMT), cell adhesion, and tumor–stroma communication (Fig. 3B). This network establishes ac⁴C as a “programmable metastatic layer” governing phenotypic plasticity and systemic dissemination.

##### EMT and phenotypic plasticity

NAT10-mediated ac⁴C dynamically remodels cytoskeletal organization and EMT-related signaling pathways. In ESCC, Wei et al. showed that tRNA ac⁴C enhances EGFR codon-biased translation, triggering EMT and promoting invasive migration [74]. In cervical cancer, Long et al. demonstrated that ac⁴C stabilization of *HNRNPUL1* mRNA drives cell migration and invasion [75]. In gastric cancer, Chen et al. found that NAT10-mediated ac⁴C modification stabilizes TNC mRNA and enhances its translation, leading to TNC protein overexpression, which activates FAK/AKT/MAPK signaling as well as TGF-β1, thereby promoting epithelial–mesenchymal transition (EMT) and metastasis [76]. Consistently, Ma et al. demonstrated that NAT10 depletion reverses EMT by restoring E-cadherin while suppressing N-cadherin/Vimentin and attenuating TGF-β/Smad and Wnt/β-catenin pathways, confirming its essential role in maintaining mesenchymal plasticity [77].

##### Adhesion, extravasation, and metastatic colonization

Beyond EMT, NAT10-mediated ac⁴C enhances metastatic efficiency through the adhesion–extravasation–colonization axis. In gastric cancer liver-metastasis models, Chen et al. showed that ac⁴C stabilization of *KLF5* mRNA upregulates integrin αV and other adhesion molecules, facilitating tumor–endothelium interactions and pre-adapting tumor cells for colonization in hepatic niches [78].

##### Tumor–stroma and immune microenvironment remodeling

NAT10-driven ac⁴C also influences the tumor microenvironment (TME) through reciprocal signaling loops involving cytokines, fibroblasts, and immune cells. Liu et al. demonstrated that GLMP-ac⁴C activates MAPK/ERK signaling, inducing secretion of IL-6, CXCL8, and CCL2, which recruit M2 macrophages, regulatory T cells (Tregs), and activate cancer-associated fibroblasts (CAFs) to remodel the extracellular matrix (ECM) [79]. In HCC, Pan et al. revealed that NAT10-ac⁴C modification of *HSP90α* establishes an ER stress–adaptive feedback loop, promoting survival under proteotoxic stress while enhancing metastasis and drug resistance [80].

#### Metabolism: ac⁴C modification and reprogramming of energy metabolism

Metabolic reprogramming is a hallmark of cancer, enabling tumor cells to sustain rapid proliferation and survive in nutrient- and oxygen-limited environments. Emerging studies reveal that NAT10-mediated N⁴-acetylcytidine (ac⁴C) modification constitutes a pivotal post-transcriptional mechanism that enhances glycolysis, coordinates metabolic–immune crosstalk, and promotes tumor progression (Fig. 3C).

##### ac⁴C regulation of glycolytic enzymes and immunometabolic rewiring

In several cancers, NAT10 directly modifies and stabilizes glycolysis-related mRNAs, thereby amplifying the expression of key metabolic enzymes. In triple-negative breast cancer (TNBC), Li et al. demonstrated that NAT10-catalyzed ac⁴C modification of *JunB* mRNA enhances *JunB* protein synthesis, which transcriptionally upregulates HK2 and LDHA, reinforcing glycolytic flux [81]. The consequent lactate accumulation not only sustains tumor metabolism but also induces immunosuppressive cytokine secretion, thereby impairing antitumor immune responses and accelerating malignant progression.

Similarly, in cervical cancer, Chen et al. reported that NAT10-dependent ac⁴C stabilization of *FOXP1* mRNA promotes *FOXP1* upregulation and glycolytic activation, leading to PD-L1 induction and CD8⁺ T-cell dysfunction, establishing a dual metabolic and immune-evasive phenotype [82]. Together, these findings reveal that ac⁴C-driven glycolysis is both an energetic driver and an immunoregulatory mechanism, coupling cellular metabolism with immune escape.

##### Metabolic feedback activation of NAT10

Intriguingly, NAT10 itself is subject to glycolysis-driven positive feedback, amplifying tumor metabolic adaptability [83, 84]. In gastric cancer, Wang et al. found that high glucose levels induce NAT10 expression, which in turn enhances HK2 transcription via ac⁴C modification, forming a metabolic–epigenetic positive feedback loop that accelerates tumor initiation and progression [83]. In a complementary mechanism, Yang et al. showed that NAT10-mediated ac⁴C stabilization of *SEPT9* mRNA promotes its interaction with HIF-1α, preventing PHD2-mediated degradation [84]. The resulting HIF-1α accumulation upregulates HK2 and LDHA, further driving glycolysis and reinforcing NAT10 expression, thereby creating a self-sustaining metabolic circuit [84].

Beyond glycolysis, NAT10-ac⁴C also enhances anabolic capacity. In osteosarcoma, Li et al. and Zou et al. demonstrated that NAT10/ac⁴C-mediated stabilization of *PFKM*, *LDHA*, and *ATF4* promotes glycolysis and asparagine biosynthesis, supporting energy generation, biomass accumulation, and metastatic competence [85, 86].

#### Cell survival and death resistance

NAT10, a stress-responsive RNA N⁴-acetylcytidine (ac⁴C)–modifying enzyme, functions as a central hub that enables cancer cells to sustain stress-adaptive survival and resist apoptosis and therapy-induced cytotoxicity. Rather than acting as a direct apoptosis suppressor, NAT10 stabilizes a subset of stress-adaptive mRNAs—including *HSP90AA1*, core unfolded protein response (UPR) regulators, and pro-survival effectors—through ac⁴C modification [80, 87]. These modifications remodel the translational landscape and proteostasis network, allowing tumor cells to delay cell death and acquire resistance to chemotherapy, targeted therapy, and immunotherapy. Beyond its RNA-modifying function, NAT10 orchestrates a broader stress integration system that links the UPR, mitochondrial metabolism, glycolysis, and drug-resistance transcriptional programs into a unified survival network.

##### Regulation of apoptosis and DNA damage response

NAT10 fine-tunes apoptotic thresholds through multi-layered mechanisms. Its inhibition markedly amplifies ER stress and activates mitochondrial apoptosis, as evidenced by upregulated GRP78, CHOP, PERK, phosphorylated eIF2α, and cleaved Caspase-3, accompanied by reduced Bcl-2 expression [87]. Under chemotherapeutic pressure, NF-κB activation upregulates NAT10, which installs ac⁴C modifications on DNA repair genes such as *XRCC1*, *PARP1*, and *RAD51*, thereby enhancing repair efficiency and suppressing apoptosis [88].

Pharmacologic inhibition of NAT10 using Remodelin disrupts rRNA processing and induces nucleolar stress, triggering p53-dependent apoptotic signaling [89]. Remodelin also reduces *ERRFI1* mRNA stability, releasing EGFR from inhibition and initiating a feedback activation loop, while concurrently suppressing PI3K/AKT survival signaling in colorectal cancer [89]. These findings establish NAT10 as a stress-contingent apoptotic modulator that simultaneously preserves genome integrity and suppresses death signaling.

##### Suppression of autophagy and ferroptosis

Beyond apoptosis, NAT10 acts as a broad-spectrum suppressor of non-apoptotic cell death pathways. By installing ac⁴C on *SGK2* mRNA, NAT10 suppresses autophagy via inhibition of the EZH2/H3K27me3/GABARAP axis, thereby preserving metabolic resources under nutrient deprivation [90]. Meanwhile, NAT10-dependent stabilization of SLC7A11 mRNA enhances glutathione synthesis and reduces reactive oxygen species (ROS) accumulation, protecting cells from ferroptotic lipid peroxidation [91]. These dual actions underscore NAT10’s capacity to coordinate cell survival across multiple death checkpoints.

##### Cytoplasmic translocation and stress-induced feedback loops

Under metabolic or proteotoxic stress, NAT10 translocates from the nucleolus to the cytoplasm, where it facilitates the recruitment of target mRNAs into stress granules, promoting transcript stabilization and translational pausing. Positive feedback circuits reinforce this adaptive state—ac⁴C-modified *HSP90AA1* mRNA mitigates ER stress, while the NAT10–HSP90α axis enhances chaperone function and drug resistance [80, 92]. Moreover, chemotherapy-induced NAT10 acetylation of ATP citrate lyase (ACLY) increases nuclear acetyl-CoA availability, driving histone hyperacetylation and transcription of drug-resistance genes [93]. These interlocking loops establish NAT10 as a nexus between RNA modification and chromatin regulation during stress adaptation.

Through coordinated RNA ac⁴C modification and protein acetylation, NAT10 fine-tunes multiple survival programs—mitigating apoptosis, suppressing autophagy and ferroptosis, and integrating UPR, DNA repair, and transcriptional plasticity (Fig. 3D). Acting as a “stress rectifier,” NAT10 allows tumor cells to postpone death, maintain proteostasis, and recover from cytotoxic stress. This multi-modal regulation underscores ac⁴C as a critical determinant of cell-fate plasticity and therapy resistance, and positions NAT10 as a strategic therapeutic target for sensitizing tumors to metabolic and genotoxic stress.

#### Therapy resistance: ac⁴C as a translational and stress-adaptation buffer

Among cytidine RNA modifications, ac⁴C appears particularly well positioned to support therapy resistance because of its central role in translational control, proteostasis, and stress adaptation. Increasing evidence indicates that NAT10-mediated ac⁴C modification enables tumor cells to survive chemotherapy, targeted therapy, and immunotherapy by reinforcing DNA repair, UPR buffering, metabolic resilience, and immune suppression.

Under chemotherapeutic stress, NF-κB-driven NAT10 induction promotes ac⁴C deposition on DNA repair transcripts, including *XRCC1*, *PARP1*, and *RAD51*, thereby enhancing repair capacity and suppressing apoptosis [86]. NAT10 also stabilizes stress-adaptive transcripts such as HSP90AA1, coordinates UPR-associated survival programs, and supports stress granule–mediated mRNA preservation, collectively postponing lethal proteotoxic collapse [78, 90]. In addition, NAT10 suppresses alternative cell death pathways by restraining autophagy and ferroptosis, further broadening its protective role under treatment conditions [88, 89].

Importantly, NAT10 also contributes to immune resistance, as its inhibition restores type I interferon signaling, improves T-cell functionality, and sensitizes tumors to immune checkpoint blockade [94, 95]. These data position ac⁴C not merely as a general oncogenic mark, but as a translational and stress-adaptation buffer that enables malignant cells to persist during therapeutic challenge.

### Role of ac⁴C in tumor microenvironment

The N⁴-acetylcytidine (ac⁴C) modification has emerged as an epitranscriptomic switch that integrates metabolic, inflammatory, and immune cues into a coordinated program of immune-suppressive homeostasis. Accumulating evidence reveals that ac⁴C reprogramming—predominantly mediated by the RNA acetyltransferase NAT10—extends far beyond intrinsic tumor cell regulation. It orchestrates macrophage polarization, T-cell exhaustion, and immune evasion, collectively shaping a refractory tumor immune microenvironment (TIME) and driving secondary resistance to immune checkpoint blockade (ICB).

#### T-cell axis: ac⁴C and T-cell exhaustion

In glioblastoma (GBM), Waibl Polania et al. demonstrated that antigen presentation by tumor-associated macrophages (TAMs) induces chronic TCR stimulation, initiating a metabolic–transcriptional exhaustion trajectory in T cells. This process is characterized by TCF1 downregulation and TOX/NR4A upregulation, accompanied by impaired glycolysis and mitochondrial respiration, promoting the transition from progenitor-exhausted (Tex_prog) to terminally exhausted (Tex_term) states and reducing responsiveness to PD-1/PD-L1 blockade [94].

Expanding this framework, Sun et al. found that TCR activation upregulates c-JUN, which transcriptionally induces NAT10, leading to ac⁴C modification of *Myc* mRNA and enhanced translational efficiency [95]. This NAT10–ac⁴C–Myc axis supports rapid clonal expansion and sustains effector T-cell activity under high metabolic demand. Conversely, NAT10 inhibition disrupts the MYC–CDK2/DNMT1 cascade, elevates intracellular double-stranded RNA (dsRNA) levels, and activates type I interferon responses, thereby amplifying CD8⁺ T cell–mediated antitumor immunity [96].

In nasopharyngeal carcinoma (NPC), Xie et al. reported that NAT10 overexpression elevates global ac⁴C levels and stabilizes CEBPG, DDX5, and HLTF transcripts. Elevated DDX5 upregulates HMGB1 secretion, which recruits immunosuppressive cells, impairs antigen presentation, and induces CD4⁺/CD8⁺ T-cell dysfunction and apoptosis, establishing an immune-suppressive TIME and promoting PD-1 resistance [97]. Importantly, HLTF transcriptionally activates NAT10, forming a self-reinforcing HLTF–NAT10 feedback loop. Pharmacologic inhibition of NAT10 with Remodelin abrogates ac⁴C deposition, disrupts the DDX5/HMGB1 axis, restores effector T-cell functionality, and enhances PD-1 blockade efficacy [97].

Collectively, these findings identify ac⁴C as a central molecular interface linking RNA modification to T-cell exhaustion, sustaining immune dysfunction within the tumor microenvironment.

#### **Macrophage axis**: ac⁴C and immunosuppressive polarization

Within the myeloid compartment, ac⁴C modification reprograms macrophage metabolism and phenotype. Jin et al. revealed that esophageal squamous cell carcinoma (ESCC) cells transfer NAT10 via exosomes to macrophages, where it catalyzes ac⁴C modification of *FASN* mRNA. This stabilizes *FASN*, enhancing lipid metabolism, triglyceride accumulation, fatty acid oxidation (FAO), and mitochondrial respiration, thereby promoting M2-like polarization and diminishing PD-1 therapy efficacy [98].

Similarly, Chen et al. demonstrated that NAT10 installs ac⁴C on CXCL2 and KLF5 transcripts, promoting M2 recruitment and polarization while inducing Oncostatin M (OSM) secretion, which activates STAT3-dependent NAT10 transcriptional feedback, reinforcing the immunosuppressive circuit [78]. In intrahepatic cholangiocarcinoma (ICC), Cai et al. found that NAT10 upregulates CCL2 to recruit M2 macrophages, further shaping an immune-suppressive niche [99].

Intriguingly, host nutritional and metabolic status also modulates TAM behavior. Zhang et al. reported that amino acid or protein restriction activates the Rag GTPases–GATOR1–FLCN nutrient-sensing axis and the mTORC1–TFEB/TFE3 pathway, enhancing lysosomal biogenesis and phagocytic metabolism, which in turn augments macrophage antitumor potential [34]. These findings highlight that ac⁴C-mediated lipid metabolism rewiring functions as a pivotal determinant of macrophage polarization and immunotherapy resistance.

**Fig. 4:**
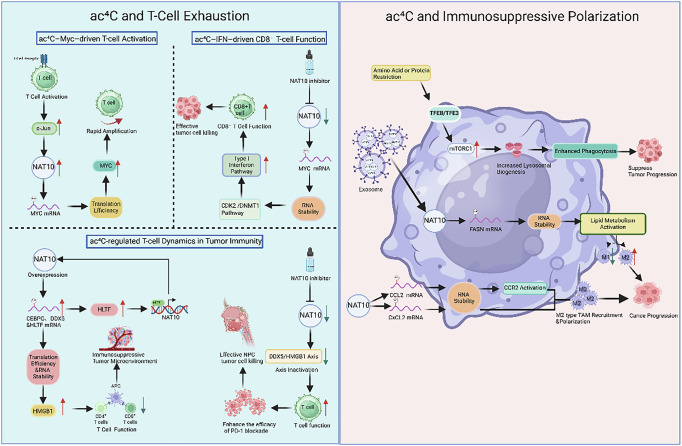
ac⁴C modification reshapes tumor immunity by regulating T-cell function and macrophage polarization. (Left panels) ac⁴C regulation of T-cell activation, exhaustion, and antitumor immunity. ac⁴C modification, mediated by NAT10, modulates T-cell function through both MYC-dependent activation and interferon-driven CD8⁺ T-cell responses. During T-cell activation, c-JUN–induced NAT10 enhances ac⁴C modification of MYC mRNA, increasing its translational efficiency and promoting rapid T-cell expansion. In parallel, ac⁴C influences type I interferon signaling and the CDK2/DNMT1 pathway, shaping CD8⁺ T-cell effector function. Dysregulated NAT10 activity stabilizes transcripts such as CEBPG, DDX5, and HLTF, promoting HMGB1-mediated immunosuppressive signaling and contributing to T-cell dysfunction and exhaustion within the tumor microenvironment. Pharmacologic inhibition of NAT10 disrupts the DDX5/HMGB1 axis, restores T-cell activity, and enhances the efficacy of PD-1 blockade. (Right panel) ac⁴C-mediated regulation of macrophage polarization and immunosuppressive tumor microenvironment. In the myeloid compartment, NAT10-dependent ac⁴C modification promotes tumor-associated macrophage (TAM) polarization toward an immunosuppressive M2-like phenotype. Tumor-derived exosomal NAT10 enhances ac⁴C modification of transcripts such as FASN, CCL2, and CXCL2, increasing RNA stability and activating lipid metabolism programs, including fatty acid oxidation and mitochondrial activity. These changes support CCR2 signaling, TAM recruitment, and M2 polarization. In parallel, nutrient-sensing pathways (e.g., mTORC1–TFEB/TFE3) regulate lysosomal biogenesis and phagocytic activity, further shaping macrophage function. Collectively, these mechanisms establish a metabolically reprogrammed, immunosuppressive microenvironment that promotes tumor progression.

Despite significant advances, several key questions remain unresolved. It is unclear whether ac⁴C modification is a causal driver or a secondary amplifier of T-cell exhaustion and M2 polarization (Fig. 4). The hierarchical relationships among its downstream effectors (e.g., *FASN*, *CXCL2*, *KLF5*, *DDX5*, *HMGB1*) and established immunosuppressive mediators—such as lactate, kynurenine, phosphatidylserine, and PD-L1—remain to be fully elucidated. Future studies combining single-cell multi-omics, conditional genetic models, and spatial transcriptomics will be critical to define the causal hierarchy of ac⁴C signaling within the TIME. Preclinical testing of temporal sequencing strategies—for example, ac⁴C inhibition followed by immune checkpoint blockade—may uncover superior therapeutic synergies compared with concurrent administration. Ultimately, these approaches could establish ac⁴C-targeted modulation as a novel immune re-sensitization strategy in refractory cancers.

## Therapeutic targeting of RNA modifications

### Inhibitory strategies targeting m⁵C

As the principal methyltransferase catalyzing 5-methylcytidine (m⁵C) modification, NSUN2 has been validated as a bona fide oncogenic driver across colorectal, hepatocellular, bladder, and breast cancers, rendering it an attractive therapeutic target. Recent chemical-biology efforts have initiated small-molecule inhibitor discovery. Structure-based virtual screening identified the candidate compound Nsun2-i4, which exhibits potent antiproliferative activity in colorectal cancer cells (SW480 IC₅₀ ≈ 56 µM; HT29 IC₅₀ ≈ 46 µM) with minimal in vivo toxicity in mice, supporting the druggability of NSUN2 [35]. However, systematic structure–activity relationship (SAR) data and comprehensive pharmacokinetic/toxicologic profiles remain limited. Future development should emphasize crystal-structure elucidation, structure-based drug design (SBDD), high-throughput screening, and optimization for selectivity and metabolic stability. In parallel, gene-silencing modalities such as siRNA or antisense oligonucleotides (ASOs) may provide transitional tools to validate target dependence before small-molecule optimization.

At the reader level, disruption of m⁵C-RNA interactions involving YBX1 or ALYREF offers an orthogonal strategy to abrogate post-transcriptional stabilization and nuclear export of oncogenic transcripts. Yet these approaches remain confined to mechanistic proof-of-concept studies, with no agents advancing to preclinical development.

Beyond direct enzyme inhibition, indirect targeting of m⁵C-linked immune and metabolic circuits is emerging as a promising avenue. For example, NSUN2-dependent maintenance of global m⁵C levels stabilizes TREX2, facilitating dsDNA clearance and suppressing the cGAS–STING–IFN pathway; conversely, NSUN2 inhibition relieves this intrinsic immunosuppressive loop, potentially enhancing immunotherapy responsiveness [48]. Collectively, these findings support a multimodal therapeutic framework, integrating m⁵C writer/reader inhibition with metabolic or immune modulation to achieve durable anticancer efficacy.

### Inhibitory strategies targeting ac⁴C

The N⁴-acetylcytidine (ac⁴C) modification, catalyzed by the acetyltransferase NAT10, promotes tumor progression through metabolic reprogramming, epithelial–mesenchymal transition (EMT), and immune evasion, and has therefore emerged as a multifunctional oncogenic target.

Remodelin, the first and most extensively studied NAT10 inhibitor, demonstrates multifaceted antitumor activity. Liu W et al. reported that Remodelin significantly suppresses tumor growth, but only in immunocompetent mice—not in nude mice—indicating that NAT10’s tumor-promoting function depends substantially on immune modulation rather than cell-autonomous proliferation [96]. Li G et al. found that Remodelin increases surface CTLA-4 expression on T cells, and its combination with anti-CTLA-4 antibody synergistically enhances T-cell activation and suppresses tumor progression [81]. Xie R et al. demonstrated that NAT10 knockout or Remodelin treatment induces γH2AX accumulation and apoptosis, thereby restraining bladder cancer growth [97]. Dalhat M.H. et al. further observed that Remodelin reprograms mitochondrial fatty-acid metabolism ( ↓ FAO, ↑ ROS), impairing cancer-cell survival and migration [100]. In addition, Guo et al. showed that Remodelin reverses EMT (E-cadherin ↑, N-cadherin/Vimentin ↓), delaying NSCLC progression and metastasis [101].

Beyond Remodelin, next-generation NAT10 inhibitors are under development. New agents that block K823 lysine 2-hydroxyisobutyrylation (Khib) destabilize NAT10 and suppress the NAT10–ac⁴C–NOTCH3 axis, thereby restraining metastasis [102]. In silico screening has also identified Paliperidone and AG-401 as candidate inhibitors targeting the NAT10–ac⁴C–ATF4–ASNS–Asn metabolic pathway, effectively suppressing osteosarcoma proliferation and metastasis in vitro and in vivo [86].

Overall, inhibition of NAT10 — either pharmacologically or genetically — offers a multidimensional anticancer strategy, simultaneously suppressing tumor proliferation, impairing metastasis, reprogramming metabolism, and re-sensitizing tumors to immune attack. As the ac⁴C field advances, systematic medicinal-chemistry optimization, pharmacodynamic biomarkers, and rational combination designs will be key to translating these mechanistic insights into clinically actionable RNA-modification therapies.

To better contextualize the translational landscape, current therapeutic strategies targeting m⁵C- and ac⁴C-related pathways are summarized in Table 1, and representative evidence linking cytidine RNA modification pathways to responses to existing anticancer therapies is summarized in Table 2. Importantly, the therapeutic rationale for targeting cytidine RNA modifications may depend less on whether they initiate tumorigenesis and more on whether tumors become selectively dependent on these pathways for stress adaptation, immune evasion, or treatment tolerance.

**Table 1:** Therapeutic strategies targeting m⁵C and ac⁴C RNA modification pathways in cancer.

Modification	Target/Axis	Inhibitor/Strategy	Cancer	Model	Main effect	Developmental status
m⁵C	NSUN2	Nsun2-i4	CRC	cell + mouse	Proliferation ↓	preclinical
	NSUN2	siRNA / ASO	multiple	conceptual / experimental	m⁵C-dependent oncogenic signaling ↓	early-stage
	YBX1 / ALYREF	reader disruption	multiple	mechanistic only	destabilize oncogenic RNAs	exploratory
	NSUN2–TREX2–cGAS/STING	indirect targeting	CRC / immune context	mechanistic	IFN ↑/ immunotherapy sensitization	exploratory
ac⁴C	NAT10	Remodelin	CRC / bladder / NSCLC / immune models	cell + mouse	proliferation↓ / apoptosis ↑ / EMT reversal / immune sensitization	preclinical
	NAT10	genetic knockout / knockdown	multiple	cell + mouse	Growth ↓ / therapy sensitivity ↑	preclinical
	NAT10 K823 Khib axis	Khib-targeting strategy	metastasis models	cell + mouse	destabilize NAT10 / metastasis ↓	preclinical
	NAT10–ATF4–ASNS	Paliperidone / AG-401	osteosarcoma	cell + mouse	metabolic adaptation ↓ / progression ↓	preclinical

This table summarizes current therapeutic approaches targeting cytidine RNA modification pathways in cancer, including direct inhibition of m⁵C-related enzymes (such as NSUN2) and ac⁴C-related regulators (primarily NAT10), as well as indirect strategies that disrupt downstream metabolic, stress-adaptive, or immune-regulatory circuits. For each strategy, representative inhibitors or perturbation modalities, associated cancer types, experimental models, principal antitumor effects, and current developmental status are listed. Collectively, these studies support the emerging druggability of the cytidine epitranscriptome and highlight its translational potential in precision oncology.

**Table 2:** Evidence linking m⁵C and ac⁴C pathways to therapeutic response and resistance in cancer.

Modification	Regulator	Therapy	Cancer	Mechanistic link	Effect on therapy response	Translational implication
m⁵C	NSUN2	Immunotherapy	CRC	TREX2 stabilization suppresses cGAS/STING	promotes resistance	combined with ICB
	YBX1	Chemotherapy	GC	stabilizes survival transcripts	promotes resistance	potential combination target
	TRDMT1 / DNMT2	DNA-damaging therapy	multiple	promotes DNA repair / HR	resistance	radiosensitization / chemosensitization
	NSUN2 / YBX1	Metabolic therapy	HCC / CRC	stabilizes PKM2 / ENO1/ glycolysis	adaptive resistance	combined with glycolysis inhibitors
ac⁴C	NAT10	Immunotherapy	NPC / immune model	DDX5/HMGB1 / T-cell dysfunction	PD-1 resistance	combine NAT10 inhibition + ICB
	NAT10	Anti-CTLA-4	Immune model	Remodeling enhances T-cell activation	improved response	combinatorial immunotherapy
	NAT10	Cisplatin / chemotherapy	bladder	XRCC1 / PARP1 / RAD51 stabilization	chemoresistance	NAT10 as chemosensitization target
	NAT10	Radiotherapy / stress therapy	multiple	UPR / proteostasis / stress survival	adaptive resistance	radiosensitization
	NAT10	Metabolic therapy	osteosarcoma / gastric	glycolysis / ATF4-ASNS / HIF1α	metabolic persistence	combined with metabolic blockade

This table summarizes representative evidence showing how m⁵C and ac⁴C regulatory pathways influence responses to existing cancer therapies, including chemotherapy, radiotherapy, targeted therapy, and immunotherapy. Listed examples illustrate how cytidine RNA modifications regulate drug sensitivity through mechanisms such as DNA repair, stress adaptation, metabolic reprogramming, immune checkpoint modulation, and cell death resistance. These findings position m⁵C and ac⁴C not only as mechanistic drivers of malignancy but also as potential predictive biomarkers and combination-therapy targets.

## Conclusions and perspectives

Cytidine RNA modifications—including 5-methylcytidine (m⁵C) and N⁴-acetylcytidine (ac⁴C)—constitute a central regulatory layer that integrates transcriptional, metabolic, and immune programs to sustain malignant progression. At the tumor-cell level, these modifications enhance mRNA stability, translation efficiency, and metabolic reprogramming, creating self-reinforcing feedback loops that underpin proliferation, invasion, and therapy resistance.

m⁵C, deposited by the NSUN family and DNMT2/TRDMT1 and interpreted by readers such as ALYREF and YBX1, modulates DNA repair, energy metabolism, and cell-death checkpoints. ac⁴C, catalyzed by NAT10, stabilizes key oncogenic and stress-adaptive transcripts, boosting translational output and coordinating survival under proteotoxic or metabolic stress. Functionally, m⁵C acts as a “mitochondrial switch” that enables adaptation to oxidative and energetic stress, whereas NAT10 serves as a “resilience organizer”, orchestrating cellular stress responses and proteostasis.

At the tumor-microenvironment (TME) level, both m⁵C and ac⁴C remodel immune ecosystems—driving macrophage polarization, T-cell exhaustion, and metabolic–immune coupling. Through cell type–specific feedback circuits, these modifications consolidate immunosuppression and contribute to resistance to immune checkpoint blockade. Together, the cytidine epitranscriptome functions as a master regulator linking intracellular homeostasis with extrinsic immune evasion.

A central unresolved question, however, is whether m⁵C and ac⁴C function primarily as oncogenic drivers, adaptive amplifiers of malignant states, or downstream consequences of oncogenic stress. Although some evidence supports driver-like roles, the current literature more often suggests that these modifications serve as state-stabilizing epitranscriptomic modules that reinforce pre-existing malignant programs under hypoxia, nutrient limitation, ER stress, DNA damage, or immune pressure. This distinction is especially relevant for ac⁴C/NAT10, which appears particularly enriched in stress adaptation, proteostasis, and therapy resistance, and for m⁵C, which frequently sustains metabolic rewiring and immune escape rather than acting as a classical initiating lesion. Resolving this issue will require site-specific epitranscriptomic editing, conditional genetic models, transcript-resolved perturbation, and temporal intervention strategies capable of distinguishing cause from consequence.

Despite rapid progress, major mechanistic and translational questions remain unresolved:

1. Mechanistic complexity: The downstream effectors of m⁵C and NAT10, and the critical mediators of ac⁴C-driven glycolytic rewiring, remain incompletely defined. The causal hierarchy among stress responses—autophagy, ER stress, and DNA repair—has yet to be established, and the sufficiency of individual ac⁴C or acetylation events in driving phenotype is unverified.
2. Biomarker validation: Although global m⁵C/ac⁴C levels and expression of NSUN2, ALYREF, YBX1, and NAT10 are promising biomarkers, their predictive power, temporal dynamics, and systemic safety windows require rigorous clinical validation.
3. Therapeutic selectivity: Achieving tumor-specific inhibition without perturbing physiological RNA modification remains challenging. The structural complexity of readers, potential compensation among NSUN family members, and context-dependent functions complicate inhibitor design.
4. Combination strategies: Rational integration of m⁵C/ac⁴C inhibition with metabolic modulation, radiotherapy, or immunotherapy demands optimization of timing, sequence, and dosing.
5. Clinical translation barriers: Pharmacokinetic limitations, delivery efficiency, off-target effects, and potential toxicity from global cytidine-modification suppression continue to hinder the development of safe, effective therapeutics.

Looking forward, future research should prioritize system-level integration and precision targeting of the cytidine epitranscriptome:

1. Cross-cancer network mapping: Construct integrative frameworks linking RNA modifications, metabolism, immune signaling, and stress resilience, to identify shared vulnerabilities and tumor-type-specific dependencies.
2. Spatiotemporal precision: Develop cell type-specific and temporally programmable interventions that selectively modulate m⁵C/ac⁴C in malignant or immune compartments.
3. Targeted drug design: Advance structure-guided inhibitors of NAT10 and NSUN enzymes and identify allosteric or site-specific modulators that minimize systemic toxicity.
4. Clinical standardization: Define m⁵C/ac⁴C expression signatures, integrate them into clinical-omics pipelines, and validate in prospective trials for patient stratification, therapy monitoring, and immunometabolic profiling.
5. Mechanistic–therapeutic coupling: Elucidate RNA–DNA–metabolism crosstalk influencing immunotherapy efficacy, enabling rational combination regimens that exploit synthetic vulnerabilities.

In summary, cytidine RNA modifications are not passive chemical marks but dynamic epigenetic actuators that coordinate tumor proliferation, metastasis, and immune remodeling. These findings underscore the emerging value of cytidine RNA modification pathways not only as mechanistic regulators of tumor plasticity but also as therapeutically actionable and clinically informative systems. Targeting the m⁵C/ac⁴C regulatory axes through mechanism-informed, metabolism-aware, and immune-integrated strategies holds promise for the next generation of precision cancer therapeutics.
